# Genome-wide SNPs and re-sequencing of growth habit and inflorescence genes in barley: implications for association mapping in germplasm arrays varying in size and structure

**DOI:** 10.1186/1471-2164-11-707

**Published:** 2010-12-15

**Authors:** Alfonso Cuesta-Marcos, Péter Szűcs, Timothy J Close, Tanya Filichkin, Gary J Muehlbauer, Kevin P Smith, Patrick M Hayes

**Affiliations:** 1 Department of Crop and Soil Science, Oregon State University, Corvallis, OR 97331, USA; 2 Department of Botany and Plant Sciences, University of California, Riverside, CA 92521, USA; 3 Department of Agronomy and Plant Genetics, University of Minnesota, St. Paul, MN 55108, USA

## Abstract

**Background:**

Considerations in applying association mapping (AM) to plant breeding are population structure and size: not accounting for structure and/or using small populations can lead to elevated false-positive rates. The principal determinants of population structure in cultivated barley are growth habit and inflorescence type. Both are under complex genetic control: growth habit is controlled by the epistatic interactions of several genes. For inflorescence type, multiple loss-of-function alleles in one gene lead to the same phenotype. We used these two traits as models for assessing the effectiveness of AM. This research was initiated using the CAP Core germplasm array (n = 102) assembled at the start of the Barley Coordinated Agricultural Project (CAP). This array was genotyped with 4,608 SNPs and we re-sequenced genes involved in morphology, growth and development. Larger arrays of breeding germplasm were subsequently genotyped and phenotyped under the auspices of the CAP project. This provided sets of 247 accessions phenotyped for growth habit and 2,473 accessions phenotyped for inflorescence type. Each of the larger populations was genotyped with 3,072 SNPs derived from the original set of 4,608.

**Results:**

Significant associations with SNPs located in the vicinity of the loci involved in growth habit and inflorescence type were found in the CAP Core. Differentiation of true and spurious associations was not possible without *a priori *knowledge of the candidate genes, based on re-sequencing. The re-sequencing data were used to define allele types of the determinant genes based on functional polymorphisms. In a second round of association mapping, these synthetic markers based on allele types gave the most significant associations. When the synthetic markers were used as anchor points for analysis of interactions, we detected other known-function genes and candidate loci involved in the control of growth habit and inflorescence type. We then conducted association analyses - with SNP data only - in the larger germplasm arrays. For both vernalization sensitivity and inflorescence type, the most significant associations in the larger data sets were found with SNPs coincident with the synthetic markers used in the CAP Core and with SNPs detected via interaction analysis in the CAP Core.

**Conclusions:**

Small and highly structured collections of germplasm, such as the CAP Core, are cost-effectively phenotyped and genotyped with high-throughput markers. They are also useful for characterizing allelic diversity at loci in germplasm of interest. Our results suggest that discovery-oriented exercises in AM in such small arrays may generate a large number of false-positives. However, if haplotypes in candidate genes are available, they may be used as anchors in an analysis of interactions to identify other candidate regions harboring genes determining target traits. Using larger germplasm arrays, genome regions where the principal genes determining vernalization sensitivity and row type are located were identified.

## Background

Association mapping (AM) (also known as linkage disequilibrium (LD) mapping) is a powerful and promising tool for gene detection in crop plants (reviewed by [1]). AM has been used in cultivated barley (*Hordeum vulgare *subsp. *vulgare*) (reviewed in [2]) and its wild progenitor - *H. vulgare subsp. spontaneum *[3-5]. A key consideration in AM is population structure, which may cause spurious correlations and lead to an elevated false-positive rate [6,7]. In cultivated barley there is a high level of population structure and the major factors determining it are growth habit and inflorescence type [4,8-11].

The major components of growth habit are sensitivity to vernalization and photoperiod, and these environmental signals are associated with tolerance to low temperature (reviewed by [12] and [13]). In general, maximum cold tolerance is achieved in genotypes with winter growth habit during the vegetative growth stage. Transition to a reproductive growth stage is triggered by vernalization and/or long photoperiod. In contrast, the flowering time of spring habit varieties is not accelerated by vernalization. Spring growth habit types may vary in their response to photoperiod, but they all have a limited capacity to tolerate low temperature stresses. Varieties with facultative growth habit can be either fall- or spring-planted since they are not vernalization sensitive. They can be as cold tolerant as winter types [14]. The photoperiod sensitivity of the facultative growth habit class varies, although sensitivity to short photoperiod is advantageous from the standpoint of delaying the vegetative to reproductive transition [15] and thus ensuring the maximum capacity for low temperature tolerance [16].

Three loci (*VRN-H1*, *VRN-H2 *and *VRN-H3*) interact in an epistatic fashion to determine vernalization sensitivity. Vernalization sensitivity occurs only in one of the possible allele combinations at these loci: *Vrn-H2_vrn-H1vrn-H1vrn-H3vrnH3 *[17]. Deletion of part or all of the genomic region encoding three zinc finger-CCT domain (ZCCT-H) transcription factors accounts for the vernalization-insensitive (recessive) alleles at the *VRN-H2 *locus [14,18-21]. Deletions spanning putative *cis*-elements in the intron I are the functional polymorphisms at *VRN-H1 *accounting for the vernalization-insensitive (dominant) alleles [14,21,22]. *HvFT1 *corresponds to the *VRN-H3 *locus and associations between intron I haplotypes and dominant/recessive alleles have been described [23,24].

Genes determining photoperiod response (*PPD-H1 *and *PPD-H2*) and low temperature tolerance (*FR-H1 *and *FR-H2*) were first reported as QTL. *PPD-H1 *affects flowering time under long-day conditions whereas *PPD-H2 *affects flowering under short photoperiods [15]. *HvPRR7 *encodes a pseudo-response regulator and corresponds to *PPD-H1 *[25]. The basis of the recessive late-flowering (long-day insensitivity) phenotype is a SNP mutation affecting a conserved residue in the CCT domain [25]. *HvFT3 *is the determinant of *PPD-H2 *[24,26]. Delayed flowering under short-day conditions (a recessive phenotype) is due to gene deletion [24,26,27]. *FR-H1 *is coincident with *VRN-H1*, but it is not yet resolved if cold temperature tolerance is a pleiotropic effect of the *VRN-H1 *[28]. One or more members of a cluster of *HvCBF *genes is (are) the candidate(s) for the *FR-H2 *locus [29-31].

The two-rowed and six-rowed inflorescence types refer to the number of fertile florets per rachis node in the barley spike. The presence of a recessive allele at the *VRS1 *locus is sufficient to cause the wild type two-rowed barley to become six-rowed barley (Lundqvist et al. 1997). Multiple loss-of-function mutations in the *HvHOX1 *gene (*VRS1*) cause a cessation of suppression of lateral-spikelet development and thus lead to the (recessive) six-rowed phenotype [32].

Previous attempts to use genome-wide AM to identify loci determining growth habit [4,10] have identified significant associations with SNP markers in the vicinity of some of the determinant genes but not the genes themselves. Cockram et al. [8], using a limited marker data set and passport data on growth habit found significant associations only when they used specific insertion/deletion (InDel) and SNP markers in the *VRN-H1 *and *VRN-H2 *genes. Most of the associated markers were designed based on the functional polymorphisms of *VRN-H1 *and *VRN-H2*.

In this report we describe, in essentially chronological order, the results of genotyping, phenotyping, and analyzing the resulting data from barley germplasm sets varying in size and composition. This research was conducted under the auspices of the Barley Coordinated Agricultural Project (CAP) [10]. The starting point for this project was the formation a small core collection (n = 102) of reference accessions contributed by each of the ten participating breeding programs, the CAP Core. The CAP Core was genotyped with SNP markers (primarily EST-based) implemented in three 1,536-plex oligonucleotide polymorphism arrays (OPAs) [33,34]. At the same time - in order to fully characterize genes involved in morphology, growth and development - a subset of 12 genes were re-sequenced or genotyped in the CAP Core. Functional polymorphism assays were based on re-sequencing of target genes in the germplasm array and based on these results, genotyping for specific SNPs and InDels. The CAP Core was phenotyped for inflorescence type and vernalization sensitivity under long-day greenhouse conditions. The SNP, functional polymorphism, and phenotype data were used for AM and analysis of interactions. Larger arrays of breeding germplasm were subsequently genotyped and phenotyped under the auspices of the CAP project. This provided 2,473 accessions phenotyped for inflorescence type and 247 accessions phenotyped for vernalization sensitivity. The SNP and phenotype data from the larger populations were then used for AM and analysis of interactions. Our objective was to empirically assess the effects of population size, population type, and analysis of interactions on detection of genes determining the principal germplasm groups of barley.

## Results

### Vernalization sensitivity and inflorescence type

In the greenhouse, under unvernalized and long-day photoperiod conditions, 91 of the 102 accessions flowered between 32-69 days after planting (Additional File 1). These genotypes are vernalization-insensitive and are therefore of spring or facultative growth habit. Eleven accessions did not flower within 150 days, at which point the experiment was terminated. These 11 genotypes are vernalization-sensitive and have winter growth habit. The 102 CAP Core accessions are comprised of 55 two-rowed and 47 six-rowed genotypes (Additional File 1). All of the two-rowed accessions but one (Charles) are vernalization-insensitive. In the six-rowed group, there are 37 vernalization-insensitive and 10 vernalization-sensitive genotypes.

### Re-sequencing and genotyping the *VRN-H*, *PPD-H*, *FR-H* and *VRS1* loci

Detailed information from the re-sequencing of 12 genes from the *VRN-H*, *PPD-H*, *FR-H *and *VRS1 *loci is presented in Additional files. The number of alleles and/or amplicon sizes, as well as number of haplotypes for each of the resequenced genes, is shown in Table 1. Sequences of the non-primer portion of each amplicon were deposited with GenBank and accession numbers are given in Additional File 2. Additional File 3 shows the gene-specific primers used for PCR amplification and fragment sequencing. Neighbor-joining phylogenetic cluster analyses are shown in Additional file 4. Detailed description of the results from the re-sequencing of the 12 genes can be found in Additional File 5.

**Table 1:** Summary of genotyping and re-sequencing of 11 genes determining growth habit and one gene determining inflorescence type

Locus	*VRN-H1*	*VRN-H2*	*VRN-H2*	*VRN-H2*	*VRN-H2*	*VRN-H3*	*VRS1*	*PPD-H2*	*PPD-H1*	*FR-H2*	*FR-H2*	*FR-H2*
Gene	*BM5A*	*ZCCT-Ha*†	*ZCCT-Hb*†	*ZCCT-Hc*†	*HvSNF2*	*HvFT1*	*HvHOX1*	*HvFT3*	*HvPRR7*	*HvCBF3*	*HvCBF6*	*HvCBF9*
Accessions Genotyped^(1)^	102	102	102	102	102	102	102	102	102	0	102	0
Number of alleles/amplicon sizes	5	2	2	2	2	2	6	2	2	0	2	0

Accessions re-sequenced^(2)^	102	-	-	-	30	102	102	26	30	30	30	30
Number of haplotypes	6	-	-	-	4	7	7	3	6	5	6	6
Sequence length (bp)	668	-	-	-	632	5670	1192	1467	1355	874	913	988

(1) Gel-based characterization of SNP, InDels

(2) Cloning and sequencing of a gene or putative functional domain within a gene

† *ZCCT-H *gene family members are present or absent

With specific reference to vernalization sensitivity and row-type, re-sequencing the promoter region, exon 1 and intron 1 of *VRN-H1 *from the 102 accessions revealed the five previously characterized intron 1 deletion types (Additional file 6) [14,21,22,35]. Comparison of the intron 1-deletion types and the haplotypes showed that some haplotypes have more than one deletion type. Sixty-seven accessions had complete deletions of the three tightly linked *ZCCT-H *genes at *VRN-H2*. Seventeen accessions - six with spring and 11 with winter growth habit - contained all three of the *ZCCT-H *genes. Eighteen vernalization-insensitive genotypes contained just the *c *form of the *ZCCT-H *gene family but had complete deletions of the *a *and *b *forms. Sequence alignment of the proposed functional polymorphism in *HvFT1 *intron 1 from accessions varying in growth habit revealed that the proposed functional polymorphism [23] does not account for phenotypic variation in growth habit in the CAP Core germplasm. The vernalization-sensitive cultivar Strider (EU007830), with a recessive *VRN-H3 *allele, showed the intron 1 haplotype hypothesized to lead to a dominant *VRN-H3 *allele. The alignment revealed a C/T promoter SNP (927^th ^nucleotide in EU007830), which better differentiated the dominant (T) and recessive (C) alleles at this locus. Alignment of the full length (1192 bp) *HvHOX1 *gene from the 102 genotypes revealed nine SNPs and seven haplotypes. Six of these haplotypes correspond to previously described alleles [32]. Four six-rowed accessions have a novel *VRS1 *haplotype. Because this novel recessive allele cannot be explained by simple mutation of any previously-described dominant alleles, we designate this allele *vrs1.a4 *(Additional File 7). The deduced polypeptide sequence of *vrs1.a4 *does not correspond to any of the reported recessive *VRS1 *alleles and sequencing additional promoter regions (data not shown) did not reveal causal polymorphisms that could explain the recessive (loss of function) phenotype conferred by *vrs.a4*. The 14 deficiens accessions in the germplasm array all have the *Vrs1.b2 *allele.

### Linkage Disequilibrium and Population Structure

**Figure 1:**
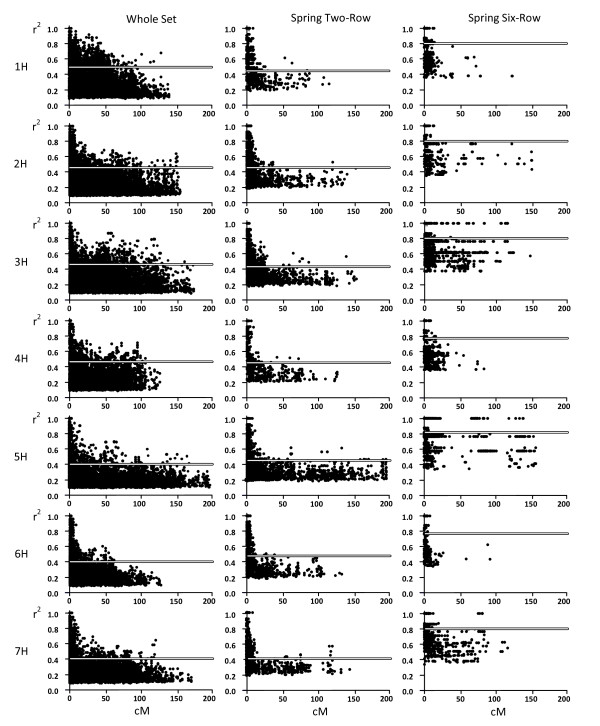
**Scatter plots of linkage disequilibrium (*r*^2^) as a function of genetic distance (cM) within chromosomes**. Significance threshold is represented as an horizontal line.

The average extent of LD is aproximately 0.7 cM when considering the entire germplasm array (Figure 1). Within the spring two-rowed group (n = 55) it is approximatetly 7 cM. Within the spring six-rowed (n = 33) it is ~ 0.2 cM. In the case of the winter six-rowed (n = 17), only pairwise comparisons whose *r*^2 ^value equals 1 have a *p*-value below than 0.001. Therefore, for this group the LD threshold was set at an *r*^2 ^value of 1 and no estimation of the extension of LD was possible.

**Figure 2:**
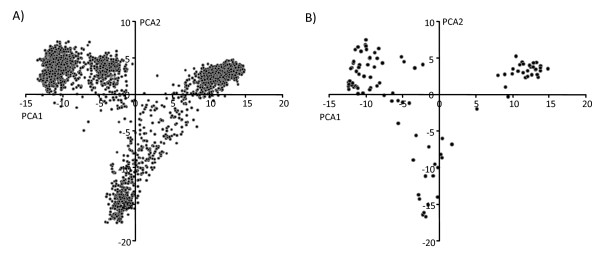
**Principal Component Analysis of the CAPI, II and III sets (A) and the CAP Core (B)**. First two axes are represented. Most of the spring 6-rowed lines are clustered in the first quadrant. Most of the spring two-rowed lines are clustered in the fourth quadrant and most of the winter 6-rowed lines are clustered in the third quadrant.

Using STRUCTURE 2.2 [36] we identified four subpopulations, the sub-population to which each accession belongs, and the percentage of admixture of each line. The four sub-populations roughly correspond to winter six-rowed, spring six-rowed, and two groups of spring two-rowed genotypes. One group of spring two-rowed genotypes is comprised of Baronesse, eight Baronesse-derived genetic stocks (BISON), and four other varieties with Baronesse in their parentage. The first two components in the Principal Components Analysis (PCA) confirmed this division by row type and growth habit, but no subgroups within the spring two-rowed genotypes were detected (Figure 2). These results are similar to those reported by [37], who performed a PCA analysis with a subset of 95 accessions of the CAP Core. There is a degree of admixture, with some accessions appearing in sub-populations that do not correspond to their growth habit or row type. For example, Charles (winter two-rowed) is clustered with the spring two-rowed; OWB-Dominant (spring two-rowed) is clustered with winter six-rowed; 88Ab536 and 88Ab536-B (winter six-rowed with Morex parentage) are clustered with spring six-rowed; and Washford, Belford, Steptoe and OWB-Recessive (spring six-rowed) are clustered with winter six-rowed.

Comparison of the first two axes of the PCA of the 102 CAP Core accessions and the PCA of the 2,473 CAP I, II and III sets shows a similar distribution of the lines (Figure 2). PCA of the latter three CAP sets shows the same pattern of division between row type and growth habit as the CAP Core. In both cases, the degree of admixture is higher among lines with the same growth habit than it is among lines with the same row type.

### Association Analysis

Growth habit and inflorescence traits are the main determinants of population structure in the CAP Core and including population structure in the model used for AM will diminish the ability to detect the genetic determinants of these traits. We therefore tested four models for AM: simple marker/trait regression (M), accounting only for the kinship of the individuals (M+K), correction by population structure (M+Q), and correction for population structure and also for the relatedness of the individuals (M+Q+K).

**Figure 3:**
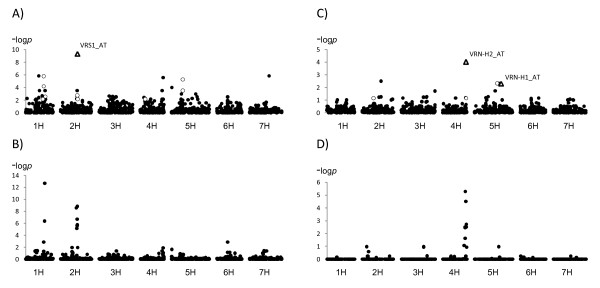
**Genome-wide association scans of A) Inflorescence type in the CAP Core; B) Inflorescence type in CAP I, II and III; C) Vernalization sensitivity in the CAP Core; D) Vernalization sensititivy in OSU CAP I, II and III**. Circles represent -log *p *values of the individual markers in consensus map position.

With each of the four analyses, we found BOPA SNPs associated with growth habit (or vernalization sensitivity) located in, or closely linked to, the principal genes determining growth habit (*VRN-H1*, *VRN-H2 *and *VRN-H3*) and inflorescence type (*VRS1*) (data not shown). The number of these significant associations - initially considered as false positives - was lower for the M+Q+K analysis compared to M+Q or M+K analyses and much lower compared with only M. Even with the M+Q+K analysis (Figure 3) without *a priori *knowledge of the roles of the genes in determining the two phenotypes, significant SNPs would not always have been the first choices for more detailed analyses: a large number of significant associations were found throughout the genome and many of these were as significant, or more significant, than those associated with the *VRN-H *and *VRS1 *loci.

The BOPA markers included 10 SNPs in the *VRN *genes that we designed based on re-sequencing: five for *VRN-H2*, two for *VRN-H1 *and three for *VRN-H3*. However, BOPA markers for *VRN-H1 *and *VRN-H2 *were not included in the association analysis because the contrasting phenotypes encoded by these genes are the consequence of gene presence vs. deletion (*VRN-H2*) or insertion/deletion (*VRN-H1*). Due to the coding of the deletion alleles as missing data, these loci fell below the quality threshold for selecting suitable markers for AM.

In order to have markers in high LD with the described functional polymorphism(s) in the *VRN-H *and *VRS1 *loci, we developed genotype assays based on our re-sequencing data and conducted a second round of AM. In this second round, we also included "synthetic markers" that summarize the dominant/recessive nature of the allele type based on the re-sequencing information and on *a priori *knowledge of gene function (VRN1_AT, VRN2_AT and VRS1_AT). The results from AM with these additional markers (Figure 3A, C) revealed highly significant associations for the synthetic markers in *VRN-H1*, *VRN-H2*, and *VRS1*. In the case of VRS1_AT for row type and VRN2_AT for flowering time, the allele type markers were the most significant (Figure 3A, C). In the case of VRN1_AT, two other markers, 10 and 20 cM from *VRN-H1*, gave similar significance.

### Analysis of Interactions

Since vernalization response is controlled by the epistatic interaction between *VRN *genes [12,14,23] and at least one other gene (*INT-C*) interacts with *VRS1 *to determine row type [38,39], we tested for interactions between significant markers detected in the M+Q+K genome-wide association analysis. For each trait we selected all markers with *p*-values below a threshold of 0.20 (after a false discovery rate multitest adjustment). Based on this criterion, 196 markers were selected for inflorescence type and 59 for vernalization sensitivity. These significant markers included the synthetic markers for allele type VRN1_AT, VRN2_AT for vernalization sensitivity and VRS1_AT for row type. For all possible two-way combinations of markers for each of the two traits, we performed likelihood ratio tests (LRTs).

For vernalization sensitivity, the interactions between five markers, out of the 1,711 possible two-way combinations, showed a remarkable increase in the -log *p *score compared with the others (Table 2). Two of the significant interacting markers were VRN1_AT and VRN2_AT. We also found similar values of -log *p *for interactions between VRN2_AT and HvZCTT_Hc_P80 with two markers on chromosome 2H: 1_0012 and 1_0624 at consensus map positions 58.9 cM and 59.2 cM respectively. Both have identical distributions of alleles within the CAP Core.

**Table 2:** Interactions with the highest LRT -log p value for vernalization sensitivity

Locus1	Chrom.	Position	Locus2	Chrom.	Position	LRT -log *p*	Rank
*ZCCT_Hc_PA*	4H	119.1	1_0624	2H	59.2	50.1	1
*ZCCT_Hc_PA*	4H	119.1	1_0012	2H	59.2	50.1	1
*VRN-H2_AT*	4H	119.1	1_0624	2H	59.2	50.0	3
*VRN-H2_AT*	4H	119.1	1_0012	2H	59.2	50.0	3
*VRN-H2_AT*	4H	119.1	*VRN-H1_AT*	5H	137.2	49.6	5
*VRN-H2_AT*	4H	119.1	1_1200	5H	117.5	31.6	6
*VRN-H2_AT*	4H	119.1	2_1027	3H	8.9	31.5	7
*ZCCT_Hc_PA*	4H	119.1	1_1200	5H	117.5	31.4	8
*ZCCT_Hc_PA*	4H	119.1	*VRN-H1_AT*	5H	137.2	30.3	9
1_1294	6H	93.7	1_1200	5H	117.5	28.8	10

Ten two-way interactions, out of the 19,110 possible, predicted perfectly the row type in the CAP Core. Of these, four interactions involved the allele type VRS1_AT. The markers involved in these interactions are on chromosomes 1H (1_0111, position 101.5 cM), 2H (1_0213, position 86.6 cM), 4H (2_0680, position 26.2 cM), and 5H (1_0899 position 59.2 cM) (Table 2). To determine if these loci (or linked loci) are determinants of the two-rowed vs. six-rowed phenotype, we examined the SNP allele type in the Oregon Wolfe Barley mapping population [34]. The parents of this population are two-rowed (OWB-dominant) and six-rowed (OWB-recessive) and the allele types at the four markers in these two accessions fits the same pattern as observed for the CAP Core and a larger population of CAP lines (see next section). However, in the OWB doubled haploid mapping population, the only locus with a perfect association with inflorescence type is *VRS1*. Six additional interactions also predicted perfectly the row type (Table 3). All of these interactions involved a marker located on 1H (2_0475, 3_0546, or 2_0550) with the same markers that interacted with VRS1_AT (1_0111 and 2_0680) or located close to them (1_0287, 2_1037, and 1_1512). Detailed information about the SNP markers detected in the interaction analysis can be found in Additional File 8.

**Table 3:** Two-way interactions that predict perfectly the row type (LRT p value is 0).

Locus 1	Chrom.	Position	Locus 2	Chrom.	Position	LRT -log *p*	Rank
*VRS1_AT*	2H	86.6	1_0213	2H	86.6	-	1
*VRS1_AT*	2H	86.6	1_0111	1H	101.5	-	1
*VRS1_AT*	2H	86.6	2_0680	4H	26.2	-	1
*VRS1_AT*	2H	86.6	1_0899	5H	59.4	-	1
2_0550	1H	88.2	1_1512	5H	59.4	-	1
3_0546	1H	92.8	1_1512	5H	59.4	-	1
3_0546	1H	92.8	1_0111	1H	101.5	-	1
3_0546	1H	92.8	2_0680	4H	26.2	-	1
2_0475	1H	92.8	1_0287	2H	85.9	-	1
2_0475	1H	92.8	2_1037	2H	88.7	-	1

### Genome-wide association analysis using larger populations

In order to assess the effects of population size on detection of genes known to determine vernalization sensitivity and inflorescence type, we conducted genome-wide association analyses for row type in the 2,575 accessions represented in the CAP Core, CAP I, CAP II, and CAP III germplasm sets (see Methods) phenotyped for this trait (Figure 3B) and the 247 winter and facultative lines from the Oregon State University barley breeding program (OSU CAP I, OSU CAPII and OSU CAP III, see Methods) phenotyped for vernalization sensitivity (Figure 3D). All these lines and accessions had been genotyped with BOPA 1 and 2.

For inflorescence type, there were significant associations on chromosomes 2H and 1H (Figure 3B), and these associations correspond to the same regions identified in the CAP Core using the allele type marker of *VRS1 *and interaction analysis. The significant markers on 2H are in the vicinity of *VRS1*; the most significant marker was 1_0213. The most significant marker on 1H was 3_1319. The allele type at each of these four loci is fixed in 99% of the two-rowed accessions (n = 1,070, 1,068, 1,065 and 1,052, respectively, out of a total of a 1,071 in the CAP I, II and III sets) (see Methods). There was no pattern for allele type in the six-rowed germplasm. For example, the alleles fixed for the two-rowed accessions are present in the following frequencies in the six-rowed accessions: 17% (1_0111), 45% (1_0213), 22% (2_0680) and 80% (1_0899).

For vernalization sensitivity, there were significant associations on chromosomes 2H, 3H, 4H and 5H (Figure 3D). These associations were found in the same genome regions as in the CAP Core, except for those found in 2H. Regarding the two loci on 2H detected in the interaction analysis in the CAP Core (1_0012 and 1_0624) we found that in 189 of the 247 accessions from this winter/facultative breeding germplasm have the same haplotype.

## Discussion

### SNP and haplotype diversity in target genes

Molecular analyses of 12 genes from seven loci known to be involved in the genetic determination of growth habit and inflorescence type - the two major traits causing strong population structuring - from a unique set of 102 barley accessions revealed high sequence diversity and complex haplotypes. For *VRN-H1*, *VRN-H3*, and *VRS1 *- the three genes where we had sequence information from the full set of 102 genotypes - we found 10, 39, and nine SNP sites, which resulted in eight, seven, and seven haplotypes, respectively. Using a subset of the core set - 26 spring, two facultative, and two winter growth habit accessions - also revealed high nucleotide diversity at six loci: *VRN-H2 *(10 SNPs and four haplotypes of *HvSNF2*), *PPD-H1 *(20 SNPs and six haplotypes of *HvPRR7*), *PPD-H2 *(7 SNPs and 3 haplotypes of *HvFT3*), and *FR-H2 (*19 SNP and five haplotypes for *HvCBF3*, 17 SNP and six haplotypes for *HvCBF6*, eight SNP and six haplotypes for *HvCBF9*). Identification of these haplotypes in marker-assisted breeding programs will require the use of multiple SNPs per gene, or alternative assays.

### Multi-locus haplotypes define growth habit classes

Even more genetic diversity was found when we considered multi-locus haplotypes of loci involved in the regulation of growth habit. Functional polymorphisms in, and epistatic interactions between, the three *VRN-H *and two *PPD-H *loci have been reported [12,14,23,27]. Based on the proposed functional polymorphisms, we determined multi-locus haplotypes of the three *VRN-H *and two *PPD-H *loci and found 16 of the possible 32-allele combinations (Additional File 1). Thirty-two out of the 85 spring growth habit cultivars had "spring" alleles at all five loci, while 41 spring growth habit genotypes had recessive (winter) *vrn-h3 *(*HvFT1*) and "spring" alleles at the other four loci. The abundance of the winter *vrn-h3 *allele in cultivated spring growth habit germplasm was unexpected since allelic variants at the *VRN-H3 *locus were previously reported only in exotic barley genotypes [17]. Seven out of 11 winter growth habit genotypes had "winter" alleles at the five loci. In total, vernalization-insensitive (spring and facultative growth habit) genotypes had 13 multi-locus haplotypes and vernalization-sensitive (winter growth habit) genotypes had three. Our results show that a large number of single-locus and multi-locus haplotypes are involved in determining flowering time and thus growth habit.

### Effect of population structure and linkage disequilibrium on association analysis

The CAP Core is a limited sample of heterogeneous germplasm. The array is comprised of accessions representing a range of growth habit, row type, usage and origin. However, this set is representative of the array of lines usually used in barley breeding programs of North America. The distribution and the number of lines in each group defined by the PCA analysis follows the same pattern in the CAP I, II and III sets and in the CAP Core (Figure 2). We used row type and growth habit (as measured by flowering time of non vernalized plants under long photoperiod) as models for genome-wide association analysis for several reasons. These phenotypes exemplify two different genetic scenarios. In the case of row type, there are multiple SNPs in the *VRS1 *gene that lead to the loss of function phenotype. The genes determining vernalization sensitivity interact epistatically and the functional basis is known. The two phenotypes can be scored unequivocally. Finally, growth habit and row type are the two traits considered world-wide to define germplasm structure in barley. Even after accounting for structure in the CAP Core, we were able to identify markers associated with the two traits because there was some degree of admixture.

The limited admixture between the three subgroups representing combinations of growth habit and row type (spring two-rowed, spring six-rowed, and winter six-rowed) is likely due to the tendency of geographically dispersed breeding programs to limit germplasm exchange. Accessions from the same structure group will have a more similar history of selection for genomic regions that control row type and growth habit than accessions from other groups. Within each structure group, accessions from any given breeding program will also tend to be more similar. As a consequence, the extent of LD is longest in germplasm of the same growth habit/row type group. However, the extent of LD within each group was reduced by the fact that accessions within each group came from different programs. The reduced estimated extension of LD in the winter six-rowed group may also be due to the small sample size (33 individuals). Barley, a self-pollinated species, is reported to have a sufficiently high level of LD that, with sufficient marker coverage, association mapping should be effective [2,11,40]. In the case of the CAP Core, we found an average extension of LD of 0.7 cM. Since the average marker density is 0.6 cM and we have markers (SNPs and/or resequencing-based assays) in the candidate genes responsible of the target traits, we would expect genome-wide association mapping to be effective.

However, the estimated extension of LD in any germplasm array must be taken cautiously, since the estimated values are averaged within each chromosome. There are individual cases of high *r*^2 ^values between distant loci (up to 100 cM in the same chromosome and even in different chromosomes) and also other cases where LD decreases abruptly within a few hundreds of bp (Figure 1).

### Genome-wide single marker association analysis

In the CAP Core we found significant associations for SNPs located in the regions of the genome where the target genes are located (Figure 3A and 3C). However, we also found other significant associations at alpha = 0.05, after multi-test adjustment, for 140 row type and seven vernalization sensitivity markers in other regions. Therefore it would be difficult to identify *VRS1 *and the *VRN-H *genes based on these results. Without *a priori *information regarding the locations of the target genes, it would be very difficult to differentiate the true associations from the spurious ones in the CAP Core.

Including "synthetic" allele type markers for *VRS1 *and *VRN-H2 *we obtained more highly significant associations than with any SNP marker. This is especially relevant in the case of VRS1, where some SNPs were located within the candidate genes (Figure 3A and 3C). In the case of *VRN-H1*, although some SNP markers from the determinant genes were also included, two other BOPA markers showed *p*-values comparable to the allele type marker (Figure 3C). However, these marker loci mapped 10 and 20 cM from *VRN-H1*. Even though these BOPA makers are in LD with the functional polymorphism they would not identify *VRN-H1 *without prior knowledge of this gene's location and function.

### Analysis of interactions

In addition to the significant interaction between the two markers for the functional polymorphisms in *VRN-H1 *and *VRN-H2*, we found a highly significant interaction for *VRN-H2 *with two markers located on the short arm of chromosome 2H (Table 2). No genes or QTLs for winter vs. spring growth habit are reported in this region, but one allele is predominant at both loci in the vernalization-sensitive accessions in the CAP Core (all 11 accessions), and OSU CAP I, II and III (96 out of 116 lines). The fact that the same allele was also present in a considerable number of vernalization insensitive (facultative) lines of the OSU CAP I, II and III (93 out of 129) suggests that this region could play a role in any of the multiple aspects of winter growth habit (e.g. low temperature tolerance or photoperiod sensitivity) since these are target traits in the OSU breeding program. A member of the CBF gene family (*HvCBF8*) cosegregates with this locus in the consensus map. Other members of the CBF gene family are implicated in low temperature tolerance [28,41,42], but no direct effect of *HvCBF8 *on cold tolerance has been reported.

Regarding row type, we found a perfect association for the interaction between allele type marker VRS1_AT with four other markers (one each on chromosomes 1H, 2H, 4H and 5H) (Table 3). All four markers are located in regions where other loci related to row type have been described [43]. These include *VRS3 *[44] on the long arm of chromosome 1H, *VRS2 *[45] and *int-b *[46] on the short arm of 5H. The marker in 2H co-locates with *VRS1 *in the consensus map [34]. The marker in 4H maps to the reported position of *INT-C *[39,47]. Alleles at these four marker loci follow a consistent pattern in the parents of the OWB population (OWB_Dominant (six-rowed) and OWB-Recessive (two-rowed)). However, there is no correlation between alleles at these four regions and the row type in the DH progeny after one cycle of recombination (even marker 1_0213 on 2H is monomorphic in this population segregating for row type). Therefore, genes in these regions are not causal for row type, but have reached a high degree of fixation in two-rowed vs. six-rowed germplasm. *INT-C *is an excellent example, where a specific dominant allele is necessary for the commercial six-rowed phenotype, but dominant or recessive alleles are found in cultivated two-rowed germplasm.

### Results of AM from larger populations

Using 2,575 lines we found significant associations in the same regions on chromosomes 2H and 1H that we detected using the CAP Core. Regarding 2H, these results confirm that the extension of LD in barley, even in highly structured germplasm, is sufficient to detect associations with markers in LD with a gene, such as *VRS1*, where different loss-of-function mutations lead to the same phenotype. The most significant markers are located in a 5 cM interval around *VRS1 *and one of them (3_0900) is located within the coding region of the gene, although it is not the marker with the highest significance (Figure 3B). On chromosome 1H we found significant markers in the same region we identified using interaction analysis and the CAP Core data. These associations may correspond to *VRS3 *[44] or to a gene, or genes, that interact with the transcription factor encoded by *VRS1*. *VRS3 *has not been characterized phenotypically or genetically in the CAP germplasm and none of the annotations for significant EST-based SNPs correspond to obvious candidate genes (HarvEST: http://www.harvest-web.org/). The CAP Core interaction analysis, and the analysis of the full set identified associations on chromosome 5H in the vicinity of *INT-B*. On chromosomes 3H, 6H and 7H significant associations were found in both analysis. There are no obvious candidate genes for these associations. Therefore, with the exception of *INT-C *(chromosome 4H), the two approaches identified the same regions.

In the case of vernalization sensitivity, using 247 accessions we found significant associations on chromosomes 4H and 5H at the same regions identified using interaction analysis based on the 102 accessions in the CAP Core (Figure 3B and 3D). These associations correspond to the locations of *VRN-H2* and *VRN-H1*, respectively. As described by [48], the most significant associations may not always correspond to candidate genes. There is no obvious candidate for the 2H associations, which differed in position between the two analyses.

## Conclusions

Small, highly structured collections, such as the CAP Core, are generally not considered ideal for association mapping. The failure to find meaningful associations between the target traits and single SNPs could be due to a number of factors including: (i) small sample size, (ii) phenotypes resulting from epistatic interactions, (iii) abrupt decay of LD around, or within, genes, (iv) multiple SNPs leading to the same phenotype, (v) the functional basis of allelic diversity is not due to SNPs, (vi) multiple haplotypes for candidate genes whose definition requires the use of multiple SNPs within each gene (vii) SNP ascertainment bias. We found that AM in the CAP Core was effective for locating genes involved in the two phenotypes - growth habit and row type - that determine the principal germplasm groups of barley only when synthetic markers based on allele re-sequencing were included and an analysis of interactions was employed. These analyses identified potential candidate interacting genes for both phenotypes. When we extended the analyses to larger populations, we identified the same genome regions. The cost of genotyping, together with the intensive computing requirements for AM, would make it difficult for most breeding programs to use this approach for detecting associations for phenotypes showing more complex inheritance. Our results suggest that breeding programs may be able to make effective use of smaller germplasm arrays for AM, provided they have a functional polymorphism data for anchor loci and use interaction analysis. The results can be useful guides for deeper exploration of significant associations in larger germplasm arrays.

## Methods

### Plant materials and phenotype evaluation

The 102 barley accessions (Additional File 1) represent key varieties, variety candidates and genetic stocks of interest to the 10 breeding programs participating in the USDA-CSREES Barley CAP http://www.barleycap.org. This germplasm collection is referred to as the CAP Core.

The vernalization sensitivity of the CAP Core was measured based on the method of [21]. All plants were grown under greenhouse conditions, without vernalization. A constant 16 h light/24 h photoperiod was provided using supplemental high intensity lighting. The temperature was maintained at 18 ± 1.5°C day and night. The number of days from seedling emergence to flowering - Zadock's scale developmental phase 9 and 49, respectively [49] - were scored for each plant. The experiment was terminated 150 days after planting, and plants that had not flowered were assigned a value of 150 days to flowering. Inflorescence type of each accession was recorded based on the number of fertile florets per rachis node and is hereafter referred to as two-rowed or six-rowed (Additional File 1).

The effects of significant markers obtained in the interaction analysis (see below, *Association mapping and interaction analysis*) were validated in other germplasm arrays of Barley CAP breeding lines. These germplasm arrays, hereafter referred to as the CAP I, II and III datasets, consist of breeding lines, elite lines, and cultivars submitted by participants in the Barley CAP http://www.barleyCAP.org. The flowering time results were validated in 247 accessions from the Oregon State University breeding program that were included in the CAP (referred as OSU CAP I, II and III). The inflorescence type results were validated using CAP I, II, and III lines from nine breeding programs (2,473). These dataset are available from The Hordeum Toolbox http://thehordeumtoolbox.org/. The Oregon Wolfe Barley Population [33], genotyped with a subset of the SNP markers used for the CAP Core BOPA1 and BOPA2 - (see below, *Genotyping*), was also used for validation of the inflorescence type results.

### Re-sequencing and genotyping the *VRN-H*, *PPD-H*, *FR-H* and *VRS1* loci

A segment of the *HvBM5A *gene (*VRN-H1*) - from the promoter CArG-box through partial intron 1 - was re-sequenced in all 102 accessions of the CAP Core. Multiple primers were designed to span all known intron 1 deletion types of *HvBM5A*. At the *VRN-H2 *locus, we genotyped all 102 accessions for the presence or absence of each of the three *ZCCT-H *genes, and a 3' UTR InDel in the *SNF2P *gene. *SNF2P *is tightly linked to the *ZCCT-H *genes [18,20]. We then re-sequenced the region flanking the 3' UTR InDel of *SNF2P *from a subset of 30 accessions (26 spring, two facultative, and two winter accessions). The lines in this subset are shown in Additional File 1. The full-length of *HvFT1 *(*VRN-H3*) was re-sequenced from ten accessions and a 1.6 kbp promoter region was re-sequenced in the remaining 96 accessions (Additional File 1). The *PPD-H1 *allele type was determined based on the CCT domain SNP in *HvPRR7 *described by [25]. In addition, we sequenced a 1.4-kbp region flanking the CCT domain in *HvPRR7 *from the same subset of 30 accessions used for deeper characterization of *SNF2P*. The *PPD-H2 *locus was genotyped as a dominant marker based on the presence/absence of the *HvFT3 *gene and the long-day insensitive alleles were re-sequenced from 26 spring accessions in the subset of 30 (Additional File 2). We re-sequenced the tightly linked *HvCBF3*, *HvCBF6*, and *HvCBF9 *genes (located at the *FR-H2 *locus) from the subset of 30 accessions as described by [30]. A 3' UTR InDel of *HvCBF6 *was genotyped in all 102 accessions. The full-length *HvHOX1 *gene (*VRS1*) was re-sequenced from all 102 accessions.

Each PCR fragment was directly sequenced as described in [50]. At least two independent PCR fragments were sequenced when a polymorphism unique to only one accession was found. Sequences of the non-primer portion of each amplicon were deposited with GenBank and accession numbers are given in Additional File 2. All genotyping PCRs were replicated at least twice to validate allele scores. Additional File 3 shows the gene-specific primers used for PCR amplification and fragment sequencing. Sequence analyses were conducted using GeneDoc version 2.6 [51] and MEGA version 4.0 [52].

### Marker development for association mapping (AM)

For AM, we converted the nucleotide polymorphism and genotyping results found in the *VRN-H*, *PPD-H*, *FR-H*, and *VRS1 *loci of the 102 genotypes into 66 binary markers. When a polymorphism site gave triple values - e.g. a SNP with three possible nucleotides or an InDel with three alleles - the minor allele was converted to missing data.

The dominant/recessive *VRN-H1 *and *VRN-H2 *allele types were determined based on [14,21,35]. Alignment of a 668 bp *VRN-H1 *(promoter region through partial intron 1) sequence revealed 10 SNPs, which we defined as markers VRN-H1_SNP1-10. According to the intron 1 deletion type we assigned dominant/recessive *VRN-H1 *alleles to the 102 accessions (marker VRN-H1_AT). Presence or absence of the *ZCCT-Ha*/*b *gene cluster determined the dominant or recessive *VRN-H2 *allele phase, respectively (marker VRN-H2_AT). The presence/absence of the *ZCCT-Hc *locus was scored as marker ZCCT-Hc_PA. The 3' UTR InDel polymorphism of *SNF2P *was scored as marker SNF2P_INDEL. Alignment of 1600 bp of the *VRN-H3 *promoter region revealed 39 SNP sites (markers VRN-H3_SNP1-39). The dominant/recessive allele phases of *PPD-H1 *and *PPD-H2 *were determined based on [25] and [24], respectively. The *PPD-H1 *and *PPD-H2 *genes were genotyped as a CAPS marker and as a presence/absence dominant PCR marker, respectively, and converted to markers PPD-H1_AT and PPD-H2_AT. From the *FR-H2 *locus we genotyped a 3' UTR InDel in the *HvCBF6 *gene and the data were used as marker CBF6_INDEL. Alignment of the 1192 bp of *HvHOX1 *(*VRS1*) - promoter region through 3' UTR - revealed nine SNPs (markers VRS1_SNP1-9). Dominant/recessive *VRS1 *allele type was determined according to [32] and converted to marker data VRS1_AT. We identified a novel allele at *VRS1 *in four six-rowed accessions (Sussex, Wysor, Nomini and Belford) that was not described in [32]. We classified these four six-rowed accessions as two-rowed for the marker VRS1_AT due to the sequence similarity of this novel six-row allele with the two-row allele (see Results). Markers developed following the dominant/recessive allele type at the *VRN-H1 *and *VRS1 *loci (VRN-H1_AT and VRS1_AT) are also called as "synthetic" markers in this publication.

Genome-wide, mostly EST-based, SNP markers were developed as described in detail in Close et al. (2009). Three 1536-plex pilot Oligonucleotide Pool Assays (POPA1, 2 and 3) were designed and used to genotype the 102 accessions using the Illumina GoldenGate BeadArray SNP detection platform [34]. Based on the re-sequencing data, we designed and included SNPs from the growth habit-related genes and *VRS1 *in POPA3 (Additional File 9). A subset of the POPA1, 2, and 3 markers were used to design Barley OPAs 1 and 2 (BOPA1 and 2) [34]. The CAP I, II, and III breeding lines were genotyped with BOPA 1 and 2. The POPA 1, 2 and 3 genotyping assays were conducted at the Southern California Genotyping Consortium at the University of California, Los Angeles by Joe DeYoung and Maricel Almonte. The BOPA assays were conducted at the USDA-ARS Regional Genotyping Laboratory at Fargo, North Dakota by Shiaoman Chao. Genetic map position of the POPA (and BOPA) markers were determined based on a consensus map employing marker segregation data from three double haploid barley mapping populations [34] (HarvEST: http://www.harvest-web.org/). SNP markers with unknown position and/or more than 10% missing data and/or Gentrain scores below 0.6 were excluded from all the analyses.

### Population structure and relatedness of individuals

Population structure in the CAP Core was determined with STRUCTURE 2.2 [36] using the linkage model and markers whose positions are defined in the consensus map (HarvEST: http://www.harvest-web.org/). For the calculation of population structure, markers with Minor Allele Frequency (MAF) below 0.05 were excluded. The program was run five independent times for each value of the maximum population number (K = 1 to K = 7) with 25,000 burn in and 25,000 MCMC iterations. For each run, an estimated logarithm of the probability (*ln Pr(X|K*) was calculated. After determining the most probable number of subpopulations, the program was run again with 100,000 burn in and 100,000 MCMC iterations in order to obtain the population structure matrix (Q matrix). This matrix defines the estimated membership of each individual to each population, expressed in proportion, and was used to correct for population structure in further analysis. Principal Components Analyses (PCA) were also used to determine population structure using SNP data and SAS v9.1 PROC PRINCOMP (SAS Institute, Cary, NC).

The Loiselle coefficient [53] was used to calculate the relatedness between individuals (relative kinship) using SPAGeDi 1.2 [54]. Two thousand polymorphic, randomly selected, SNPs from POPA1 and POPA2 were used in the analysis. Negative values of the coefficient between two individuals, which mean that the two individuals are less related than two random individuals, were set to zero. A matrix with the relative kinship coefficients (K matrix) was used to correct for relatedness of individuals in further analyses.

### Linkage disequilibrium

TASSEL 2.1 http://www.maizegenetics.net[55] was used to calculate the LD parameter *r*^2 ^and corresponding *p*-values (two-sided Fisher's exact test). For the calculation of LD, markers with Minor Allele Frequency (MAF) below 0.05 were excluded. The extent of LD was calculated for each chromosome for the whole set of 102 accessions, and also for the different groups of growth habit and inflorescence type: spring two-rowed (54 accessions), spring six-rowed (33 accessions) and winter six-rowed (17 accessions). The calculation was based on the approach described by [56]. We calculated *r*^2 ^values between all unlinked loci and, separately, *r*^2 ^values for the loci in the same chromosome. For each chromosome, *r*^2 ^with *p*-values below 0.001 were plotted against genetic distance (cM), and a second-degree smoothed loess curve with 90% confidence limits was fit using SAS PROC LOESS (SAS v9.1). The SAS macro *bctrans *[57] was used to perform a Box-Cox power transformation [58] of the unlinked *r*^2 ^values in order to approximate a normal distribution. The parametric 95th percentile of the distribution was used as a threshold to consider that LD was likely caused by genetic linkage. The intersection of the Loess curve and its confidence bands with a baseline drawn at the threshold value was considered to estimate the extent of LD, with confidence intervals, for each chromosome.

### Association mapping and interaction analysis

Genome-wide association analysis was performed using TASSEL 2.1. Four models were tested: (i) only markers as a fixed factor (M), (ii) markers as a fixed factor and the population structure defined by the Q matrix as a co-variate (M+Q), (iii) markers as a fixed factor and the genetic background as a random polygenic effect with a variance covariance structure defined by the K matrix (M+K), and (iv) markers as a fixed factor, the Q matrix as covariate and genetic background as a random polygenic effect (M'+Q+K). A False Discovery Rate multi-test adjustment [59] was performed on the results and markers that showed a *p*-value below 0.2 in the M+Q+K model were selected for an analysis of interactions. To find the interactions that best explained the phenotypic data, we performed a likelihood ratio test (LRT) subtracting the *-2 log likelihood *for the model that included the two markers being tested, their interaction and the population structure from the model that included only the population structure. Since these tests are for fixed effects parameters, *-2 log likelihood *for the models was estimated using the maximum likelihood method (ML) of SAS PROC MIXED (SAS v9.1). The asymptotic null distribution of the test statistic is a *χ*^2 ^with degrees of freedom equal to the difference in the number of fixed-effect parameters between the two models. Validation of the results of AM analysis of row type in the CAP I, II and III sets was done using TASSEL 2.1 and a M+Q+K model. Population structure was determined by means of PCA using SAS PROC PRINCOMP and relatedness of individuals was corrected using a the K matrix calculated with TASSEL 2.1.

## List of abbreviations

AM: association mapping; BOPA barley oligonucleotide pool assay; CAP: coordinated agricultural project; EST: expressed sequence tag; InDels: insertions/deletions; LD: linkage disequilibrium; MAF: minor allele frequency; OWB: Oregon Wolfe Barley; PCA: principal components analysis; POPA: pilot oligonucleotide pool assay; QTL: quantitative trait locus; SNPs: single nucleotide polymorphisms.

## Authors' contributions

ACM, PS, PMH, designed the experiment, interpreted the results and wrote the manuscript; PS scored the phenotypes; PS and TF performed re-sequencing; ACM and PS performed statistical analyses; TJC analyzed the SNP genotype data; GM and KS provided insightful interpretations of key results. All authors read and approved the final manuscript.

## Supplementary Material

Additional file 1 **Table S1**. Passport information and phenotypic characterization of the CAP Core for flowering time and inflorescence type and genotypic characterization for the main loci controlling these traits. Click here for file

Additional file 2 **Table S2**. Accession numbers of the sequences deposited with GenBank. Click here for file

Additional file 3 **Table S3**. Primers used for genotyping and/or re-sequencing VRN-H, PPD-H, FR-H, and VRS1 specific genes in the CAP Core. Click here for file

Additional file 4 **Figure S1**. Neighbor-Joining phylogenetic cluster analyses of several re-sequenced genes of the barley CAP Core set. Confidence values on the branches are based on 1000 bootstraps. For each gene, sequence length and number of lines used is indicated in the heading of each cluster. Number of genotypes per haplotype is indicated in brackets. Click here for file

Additional file 5 **Text S1**. Detailed results of re-sequencing. Click here for file

Additional file 6 **Figure S2**. Schematic representation of *VRN-H1 *intron 1 found in the barley CAP Core set (102 genotypes). Deletion types are named based on the first cultivar whose sequence was deposited in GenBank. Edges of exons 1 and 2 are indicated by flanking black boxes. Gaps represent deletions (>50 bp) relative to the full-length Strider allele. The 2.8 kb barley-wheat conserved region (dashed line) and the 436 bp vernalization critical region (dotted box) are indicated. Number of genotypes per deletion type is denoted. Click here for file

Additional file 7 **Figure S3**. Sequence alignment of *HvHOX1 *from seven accessions with different VRS1 haplotypes. Similarity is shown to four levels. Click here for file

Additional file 8 **Table S4**. Detailed information about SNP markers highly significant in the analysis of interactions in the CAP Core. Click here for file

Additional file 9 **Table S5**. POPA SNP markers developed from re-sequencing of loci related to vernalization sensitivity and inflorescence type. Click here for file
