# Supplementary material for: Genome-wide SNPs and re-sequencing of growth habit and inflorescence genes in barley: implications for association mapping in germplasm arrays varying in size and structure
Source: BMC Genomics. 2010 Dec 15;11:707. doi: 10.1186/1471-2164-11-707 (PMC3018479; doi:10.1186/1471-2164-11-707)
Supplement: Additional file 4 — Figure S1. Neighbor-Joining phylogenetic cluster analyses of several re-sequenced genes of the barley CAP Core set. Confidence values on the branches are based on 1000 bootstraps. For each gene, sequence length and number of lines used is indicated in the heading of each cluster. Number of genotypes per haplotype is indicated in brackets. [file 1471-2164-11-707-S4.PPT]

## Slide 1
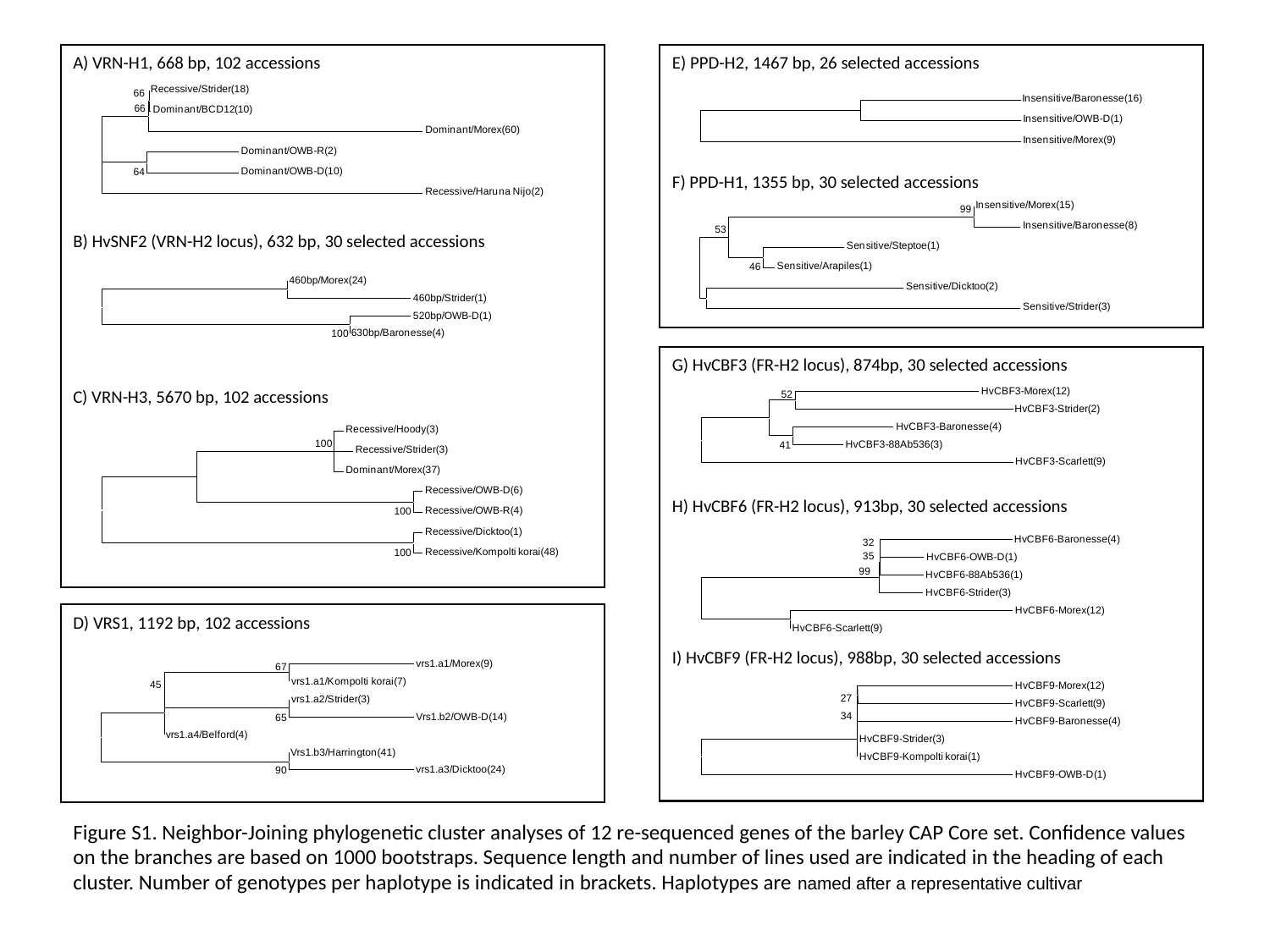

A) VRN-H1, 668 bp, 102 accessions
E) PPD-H2, 1467 bp, 26 selected accessions
F) PPD-H1, 1355 bp, 30 selected accessions
B) HvSNF2 (VRN-H2 locus), 632 bp, 30 selected accessions
G) HvCBF3 (FR-H2 locus), 874bp, 30 selected accessions
C) VRN-H3, 5670 bp, 102 accessions
H) HvCBF6 (FR-H2 locus), 913bp, 30 selected accessions
D) VRS1, 1192 bp, 102 accessions
I) HvCBF9 (FR-H2 locus), 988bp, 30 selected accessions
Figure S1. Neighbor-Joining phylogenetic cluster analyses of 12 re-sequenced genes of the barley CAP Core set. Confidence values on the branches are based on 1000 bootstraps. Sequence length and number of lines used are indicated in the heading of each cluster. Number of genotypes per haplotype is indicated in brackets. Haplotypes are named after a representative cultivar
