# Supplementary material for: Genome-wide SNPs and re-sequencing of growth habit and inflorescence genes in barley: implications for association mapping in germplasm arrays varying in size and structure
Source: BMC Genomics. 2010 Dec 15;11:707. doi: 10.1186/1471-2164-11-707 (PMC3018479; doi:10.1186/1471-2164-11-707)
Supplement: Additional file 5 — Text S1. Detailed results of re-sequencing. [file 1471-2164-11-707-S5.DOC]

*Re-sequencing and genotyping the VRN-H, PPD-H, FR-H and VRS1 loci*

Re-sequencing the promoter region, exon 1 and intron 1 of *VRN-H1* from the 102 accessions revealed the five previously characterized intron 1 deletion types [14,21,22,36]. Deletion types are named based on the cultivar whose sequence was first deposited in GenBank: seven accessions were Albacete (AY866494); 3 Maskin (DQ924860); 53 Morex (AY758233), 12 OWB-D (AY750996), 10 Triumph (AY871789) (Additional File 6) And 17 genotypes had the full length, Strider (AY750993) type intron 1 (Additional File 6). Alignment of a 668 bp fragment present in all genotypes revealed six haplotypes (Additional File 4). Comparison of the intron 1-deletion types and the haplotypes showed that some haplotypes have more than one deletion type.

Sixty-seven accessions had complete deletions of the three tightly linked *ZCCT-H* genes at *VRN-H2* (Additional File 1). Seventeen accessions − six with spring and 11 with winter growth habit − contained all three of the *ZCCT-H* genes. Eighteen vernalization-insensitive genotypes contained just the *c* form of the *ZCCT-H* gene family but had complete deletions of the *a* and *b* forms. Genotyping a 3` UTR InDel in the *SNF2P* gene gave the same three amplicon sizes that were reported by [21]. Sixteen genotypes with the dominant *VRN-H2* allele and 59 with the recessive allele had the short (540 bp) amplicon. Twenty-six genotypes with the recessive *VRN-H2* allele had the long (710 bp) amplicon. OWB-D had a unique, 620 bp long *SNF2P* amplicon (Additional File 1). Re-sequencing of the region flanking the InDel from 26 spring, two facultative, and two winter accessions revealed four haplotypes (Additional File 4).

To validate the proposed functional polymorphism in the *VRN-H3* intron 1 [23], we re-sequenced the full *VRN-H3* gene (5760 bp) from 10 genotypes (GenBank Accessions EU007825-EU007834). The dominant / recessive *VRN-H3* allele type of these accessions was classified according to [21] (Cali-sib, OWB-Dominant and Dicktoo), [23] (Dairokkaku and Tammi) and according to winter growth habit (Strider, Luca, Kompolti, Waxbar and Merlin). Sequence alignment revealed that the proposed functional polymorphism was not valid in this sample of germplasm since the vernalization-sensitive cultivar Strider (EU007830), with a recessive *VRN-H3* allele, showed the intron 1 haplotype hypothesized to lead to a dominant *VRN-H3* allele. The alignment revealed a C/T promoter SNP (927^th^ nucleotide in EU007830), which better differentiated the dominant (T) and recessive (C) alleles at this locus. We re-sequenced a 1.6 kbp promoter region flanking this “associated” SNP from the 102 accessions (Additional File 2) and found 37 genotypes with the dominant and 65 with the recessive allele (Additional File 1). While the dominant allele was conserved and had just one haplotype, six promoter haplotypes led to the recessive allele (Additional File 4).

Eighty-one accessions, including one with winter growth habit, had the CCT domain (loss of function, recessive) mutation in *PPD-H1* [26] and are therefore long-day insensitive (Additional File 1). Ten winter and 11 vernalization-insensitive accessions had the wild type, long-day sensitive (dominant) allele. Re-sequencing the 3’ end of the gene (1355 bp) from 26 spring, two facultative, and two winter growth habit accessions revealed two haplotypes for the long-day insensitive allele and four haplotypes for the sensitive allele (Additional File 4).

Eighty-three of the 85 spring growth habit accessions had the dominant (photoperiod insensitive) *HvFT3 (PPD-H2)* allele and eight of the 11 winter growth habit genotypes had the recessive (null) allele [24,27] (Additional File 1). Two spring and two facultative growth habit accessions had the recessive (short day sensitive) allele while three facultative and three winter accessions had the dominant allele. Re-sequencing the *HvFT3* gene (1467 bp) from 26 vernalization-insensitive accessions revealed additional allelic variation at the *PPD-H2* locus and revealed three short-day insensitive haplotypes (Additional File 4).

We re-sequenced the full-length *HvCBF3* (874 bp), *HvCBF6* (913 bp), and *HvCBF9* (988 bp) genes – which are tightly linked at the *FR-H2* locus – from 30 selected accessions (26 spring, two facultative, and two winter) and found 19, 17, and eight SNPs, respectively. We observed five, six and six haplotypes for *HvCBF3*, *HvCBF6* *HvCBF9*, respectively (Additional File 4). Considering all three CBF genes, twenty-five of the 26 spring growth habit genotypes formed three clusters of haplotype groups (Additional File 4) named after a representative cultivar (Morex (12), Baronesse (4), and Scarlett (9)). Winter and facultative cultivars were not clustered with the spring types for any of the CBFs. Screening a 3` UTR InDel in *HvCBF6* gave two alleles: all winter (11), facultative (6), and 57 spring growth habit accessions produced a 250 bp long amplicon, whereas 28 spring growth habit genotypes gave a 263 bp long PCR product (Additional File 1).

Alignment of the full length (1192 bp) *VRS1* gene from the 102 genotypes revealed nine SNP sites and seven haplotypes. Six of these haplotypes correspond to alleles previously described and named by [33]. There are 14 and 41 two-rowed accessions with the *Vrs1.b2* and *Vrs1.b3* alleles, respectively. There were and 16, 3, and 24 six-rowed genotypes with the *vrs1.a1*, *vrs1.a2*, and *vrs1.a3* alleles, respectively (Additional File 4). We found only two of three *vrs1.a1* haplotypes (Morex type and Soren Oomugi 19329 type) reported by [33]. Four six-rowed accessions have a novel *VRS1* haplotype. Because this novel recessive allele cannot be explained by simple mutation of any previously-described dominant alleles, we designate this allele *vrs1.a4* (Additional File 6). The deduced polypeptide sequence of *vrs1.a4* does not correspond to any of the reported recessive *VRS1* alleles and sequencing additional promoter regions (data not shown) did not reveal causal polymorphisms that could explain the recessive (loss of function) phenotype conferred by *vrs.a4*. The 14 deficiens accessions in the germplasm array all have the *Vrs1.b2* allele. Alignment of the *Vrs1.b2* allele sequence and the *Vrs1.t* allele from the deficiens Ethiopian landrace Debre_Zeit ([AB489131.1](http://www.ncbi.nlm.nih.gov/entrez/query.fcgi?cmd=Retrieve&db=Nucleotide&list_uids=224487854&dopt=GenBank&RID=3RSD88Y701S&log$=nucltop&blast_rank=76)) revealed no differences.
