# Supplementary material for: Genome-wide SNPs and re-sequencing of growth habit and inflorescence genes in barley: implications for association mapping in germplasm arrays varying in size and structure
Source: BMC Genomics. 2010 Dec 15;11:707. doi: 10.1186/1471-2164-11-707 (PMC3018479; doi:10.1186/1471-2164-11-707)
Supplement: Additional file 6 — Figure S2. Schematic representation of VRN-H1 intron 1 found in the barley CAP Core set (102 genotypes). Deletion types are named based on the first cultivar whose sequence was deposited in GenBank. Edges of exons 1 and 2 are indicated by flanking black boxes. Gaps represent deletions (>50 bp) relative to the full-length Strider allele. The 2.8 kb barley-wheat conserved region (dashed line) and the 436 bp vernalization critical region (dotted box) are indicated. Number of genotypes per deletion type is denoted. [file 1471-2164-11-707-S6.PPT]

## Slide 1
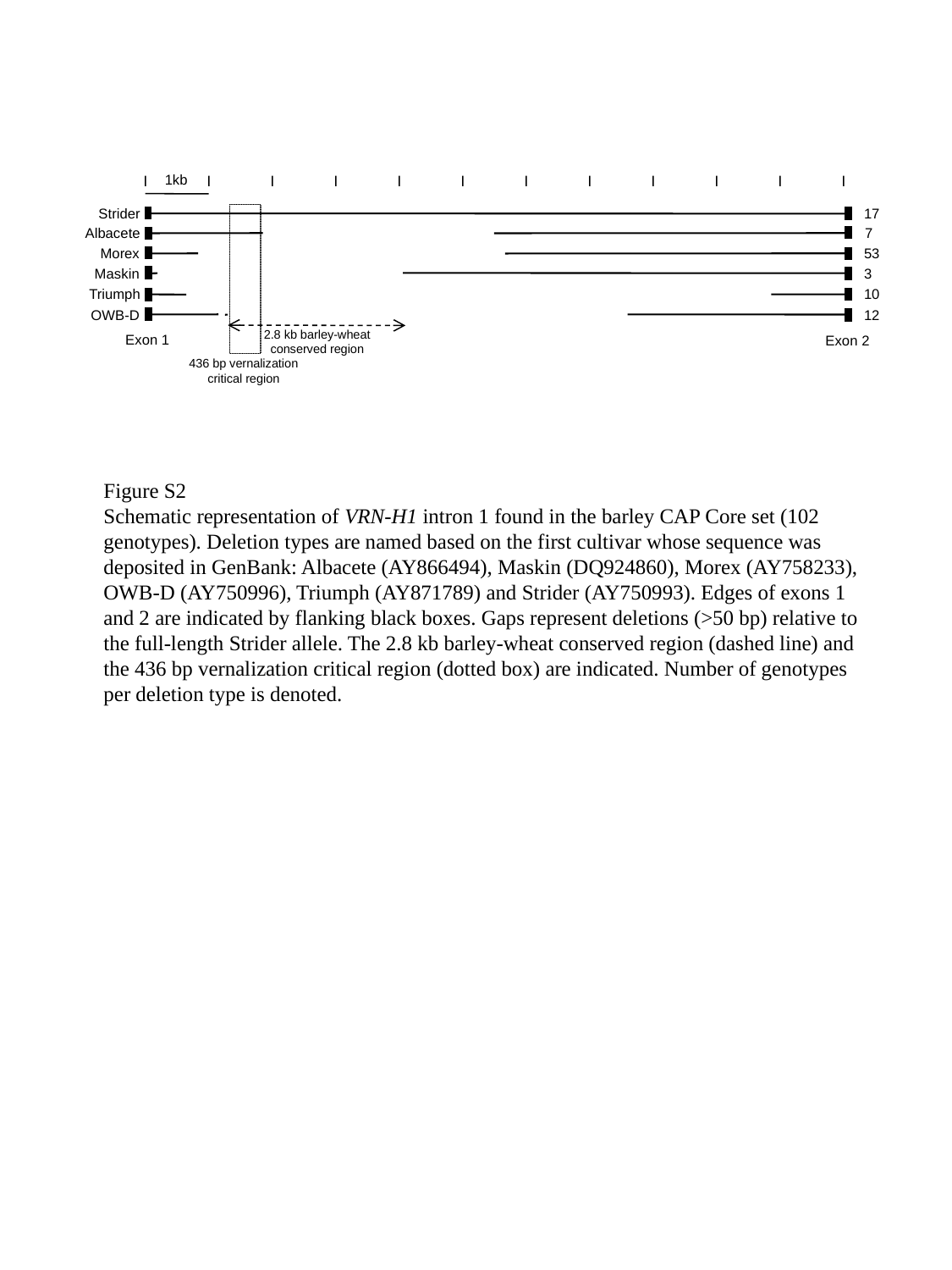

1kb
Strider
17
Albacete
7
Morex
53
Maskin
3
10
Triumph
OWB-D
12
2.8 kb barley-wheat conserved region
Exon 1
Exon 2
436 bp vernalization critical region
Figure S2
Schematic representation of VRN-H1 intron 1 found in the barley CAP Core set (102 genotypes). Deletion types are named based on the first cultivar whose sequence was deposited in GenBank: Albacete (AY866494), Maskin (DQ924860), Morex (AY758233), OWB-D (AY750996), Triumph (AY871789) and Strider (AY750993). Edges of exons 1 and 2 are indicated by flanking black boxes. Gaps represent deletions (>50 bp) relative to the full-length Strider allele. The 2.8 kb barley-wheat conserved region (dashed line) and the 436 bp vernalization critical region (dotted box) are indicated. Number of genotypes per deletion type is denoted.
