# Supplementary material for: Genome-wide SNPs and re-sequencing of growth habit and inflorescence genes in barley: implications for association mapping in germplasm arrays varying in size and structure
Source: BMC Genomics. 2010 Dec 15;11:707. doi: 10.1186/1471-2164-11-707 (PMC3018479; doi:10.1186/1471-2164-11-707)
Supplement: Additional file 7 — Figure S3. Sequence alignment of HvHOX1 from seven accessions with different VRS1 haplotypes. Similarity is shown to four levels. [file 1471-2164-11-707-S7.DOC]

**Figure S3** Sequence alignment of *HvHox1* from seven accessions with different *VRS1* haplotypes. Similarity is shown to four levels.

* 20 * 40 * 60 * 80 * 100
Hox1_1 : agCACTCAGCCGGAGCCCCGCCGGCGCTTGCCGTTGGGTACCTCTGCCGCCTATTTATATTACCCCTAGGTCTCTCCCTGGAGACACGCACTCCCCTCCTTCAACTA : 107
Hox1_30 : AGCACTCAGCCGGAGCCCCGCCGGCGCTTGCCGTTGGGTACCTCTGCCACCTATTTATATTACCCCTAGGTCTCTCCCTGGAGACACGCACTCCCCTCCTTCAACTA : 107
Hox1_38 : AgcaCTcAGcCgGAGccCCGCCGGCGCTTGCCGTTGGGTACCTCTGCCACCTATTTATATTACCCCTAGGTCTCTCCCTGGAGACACGCACTCCCCTCCTTCAACTA : 107
Hox1_86 : aGCACTCAGCCGGAGCCCCGCCGGCGCTTGCCGTTGGGTACCTCTGCCACCTATTTATATTACCCCTAGGTCTCTCCCTGGAGACACGCACTCCCCTCCTTCAACTA : 107
Hox1_95 : AgcACTCAGCCGGAGCCCCGCCGGCGCTTGCCGTTGGGTACCTCTGCCACCTATTTATATTACCCCTAGGTCTCTCCCTGGAGACACGCACTCCCCTCCTTCAACTA : 107
Hox1_96 : agCACTCAGCCGGAGCCCCGCCGGCGCTTGCCGTTGGGTACCTCTGCCACCTATTTATATTACCCCTAGGTCTCTCCCTGGAGACACGCACTCCCCTCCTTCAACTA : 107
Hox1_101 : AgcACTCAGCCGGAGCCCCGCCGGCGCTTGCCGTTGGGTACCTCTGCCACCTATTTATATTACCCCTAGGTCTCTCCCTGGAGACACGCACTCCCCTCCTTCAACTA : 107
 AGCACTCAGCCGGAGCCCCGCCGGCGCTTGCCGTTGGGTACCTCTGCCaCCTATTTATATTACCCCTAGGTCTCTCCCTGGAGACACGCACTCCCCTCCTTCAACTA

 * 120 * 140 * 160 * 180 * 200 *
Hox1_1 : GTGCTTTGCGGCCCGTGGTCCTCCTCTCGATCCAGTTCCTGAGCACACCAACAGGCAACAGAACAACCTACCGTGTCTCCCCTCCAATCTCCTCACGATCCCTTCTT : 214
Hox1_30 : GTGCTTTGCGGCCCGTGGTCCTCCTCTCGATCCAGTTCCTGAGCACACCAACAGGCAACAGAACAACCTACCGTGTCTCCCCTCCAATCTCCTCACGATCCCTTCTT : 214
Hox1_38 : gTGCTTTGCGGcCCGTGGTCCTCCTCTCGATcCAGTTCCTGAGCACACCAACAGGCAACAGAACAACCTACCGTGTCTCCCCTCCAATCTCCTCACGATCCCTTCTT : 214
Hox1_86 : GTGCTTTGCGGCCCGTGGTCCTCCTCTCGATCCAGTTCCTGAGCACACCAACAGGCAACAGAACAACCTACCGTGTCTCCCCTCCAATCTCCTCACGATCCCTTCTT : 214
Hox1_95 : GTGCTTTGCGGCCCGTGGTCCTCCTCTCGATCCAGTTCCTGAGCACACCAACAGGCAACAGAACAACCTACCGTGTCTCCCCTCCAATCTCCTCACGATCCCTTCTT : 214
Hox1_96 : GTGCTTTGCGGCCCGTGGTCCTCCTCTCGATCCAGTTCCTGAGCACACCAACAGGCAACAGAACAACCTACCGTGTCTCCCCTCCAATCTCCTCACGATCCCTTCTT : 214
Hox1_101 : GTGCTTTGCGGCCCGTGGTCCTCCTCTCGATCCAGTTCCTGAGCACACCAACAGGCAACAGAACAACCTACCGTGTCTCCCCTCCAATCTCCTCACGATCCCTTCTT : 214
 GTGCTTTGCGGCCCGTGGTCCTCCTCTCGATCCAGTTCCTGAGCACACCAACAGGCAACAGAACAACCTACCGTGTCTCCCCTCCAATCTCCTCACGATCCCTTCTT

 220 * 240 * 260 * 280 * 300 * 320
Hox1_1 : TCCCTCAGATCCGAACCGAAAGCATGGACAAGCATCAGCTCTTTGGTTCATCCAACGTGGACACGACTTTCTTCGCGGCCAATGGTACACACGACGCCGCGCGCGCC : 321
Hox1_30 : TCCCTCAGATCCGAACCGAAAGCATGGACAAGCATCAGCTCTTTGATTCATCCAACGTGGACACGACTTTCTTCGCGGCCAATGGTACACACGACGCCGCGCGCGCC : 321
Hox1_38 : TCCCTCAGATCCGAACCGAAAGCATGGACAAGCATCAGCTCTTTGGTTCATCCAACGTGGACACGACTTTCTTCGCGGCCAATGGTACACACGACGCCGCGCGCGCC : 321
Hox1_86 : TCCCTCAGATCCGAACCGAAAGCATGGACAAGCATCAGCTCTTTGGTTCATCCAACGTGGACACGACTTTCTTCGCGGCCAATGGTACACACGACGCCGCGCGCGCC : 321
Hox1_95 : TCCCTCAGATCCGAACCGAAAGCATGGACAAGCATCAGCTCTTTGATTCATCCAACGTGGACACGACTTTCTTCGCGGCCAATGGTACACACGACGCCGCGCGCGCC : 321
Hox1_96 : TCCCTCAGATCCGAACCGAAAGCATGGACAAGCATCAGCTCTTTGGTTCATCCAACGTGGACACGACTTTCTTCGCGGCCAATGGTACACACGACGCCGCGCGCGCC : 321
Hox1_101 : TCCCTCAGATCCGAACCGAAAGCATGGACAAGCATCAGCTCTTTGGTTCATCCAACGTGGACACGACTTTCTTCGCGGCCAATGGTACACACGACGCCGCGCGCGCC : 321
 TCCCTCAGATCCGAACCGAAAGCATGGACAAGCATCAGCTCTTTG TTCATCCAACGTGGACACGACTTTCTTCGCGGCCAATGGTACACACGACGCCGCGCGCGCC

 * 340 * 360 * 380 * 400 * 420
Hox1_1 : CGGTCTTTGCGCATGCGATGATGCAGCTGCAGTAGCTTCAGTTTCACCGGCCAGGACACGCATGTGATGACGTTTTTTCCATTCTGTGTTTGTATGTGCAGGCACGG : 428
Hox1_30 : CGGTCTTTGCGCATGCGATGATGCAGCTGCAGTAGCTTCAGTTTCACCGGCCAGGACACGCATGTGATGACGTTTTTTCCATTCTGTGTTTGTATGTGCAGGCACGG : 428
Hox1_38 : CGGTCTTTGCGCATGCGATGATGCAGCTGCAGTAGCTTCAGTTTCACCGGCCAGGACACGCATGTGATGACGTTTTTTCCATTCTGTGTTTGTATGTGCAGGCACGG : 428
Hox1_86 : CGGTCTTTGCGCATGCGATGATGCAGCTGCAGTAGCTTCAGTTTCACCGGCCAGGACACGCATGTGATGACGTTTTTTCCATTCTGTGTTTGTATGTGCAGGCACGG : 428
Hox1_95 : CGGTCTTTGCGCATGCGATGATGCAGCTGCAGTAGCTTCAGTTTCACCGGCCAGGACACGCATGTGATGACGTTTTTTCCATTCTGTGTTTGTATGTGCAGGCACGG : 428
Hox1_96 : CGGTCTTTGCGCATGCGATGATGCAGCTGCAGTAGCTTCAGTTTCACCGGCCAGGACACGCATGTGATGACGTTTTTTCCATTCTGTGTTTGTATGTGCAGGCACGG : 428
Hox1_101 : CGGTCTTTGCGCATGCGATGATGCAGCTGCAGTAGCTTCAGTTTCACCGGCCAGGACACGCATGTGATGACGTTTTTTCCATTCTGTGTTTGTATGTGCAGGCACGG : 428
 CGGTCTTTGCGCATGCGATGATGCAGCTGCAGTAGCTTCAGTTTCACCGGCCAGGACACGCATGTGATGACGTTTTTTCCATTCTGTGTTTGTATGTGCAGGCACGG

 * 440 * 460 * 480 * 500 * 520 *
Hox1_1 : CGCAGGGGGAGACCAGCAAGCAGAGGGCGCGGCGCAGGCGGCGGAGGTCGGC-GAGGTGCGGCGGAGGGGATGGTGACGGTGGGGAGATGGACGGAGGAGGGGACCC : 534
Hox1_30 : CGCAGGGGGATACCAGCAAGCAGAGGGCGCGGCGCAGGCGGCGGAGGTCGGC-GAGGTGCGGCGGAGGGGATGGTGACGGTGGGGAGATGGACGGAGGAGGGGACCC : 534
Hox1_38 : CGCAGGGGGAGACCAGCAAGCAGAGGGCGCGGCGCAGGCGGCGGAGGTCGGCTGAGGTGCGGCGGAGGGGATGGTGACGGTGGGGAGATGGACGGAGGAGGGGACCC : 535
Hox1_86 : CGCAGGGGGAGACCAGCAAGCAGAGGGCGCGGCGCAGGCGGCGGAGGTCGGC-GAGGTGCGGCGGAGGGGATGGTGACGGTGGGGAGATGGACGGAGGAGGGGACCC : 534
Hox1_95 : CGCAGGGGGATACCAGCAAGCAGAGGGCGCGGCGCAGGCGGCGGAGGTCGGC-GAGGTGCGGCGGAGGGGATGGTGACGGTGGGGAGATGGACGGAGGAGGGGACCC : 534
Hox1_96 : CGCAGGGGGAGACCAGCAAGCAGAGGGCGCGGCGCAGGCGGCGGAGGTCGGC-GAGGTGCGGCGGAGGGGATGGTGACGGTGGGGAGATGGACGGAGGAGGGGACCC : 534
Hox1_101 : CGCAGGGGGAGACCAGCAAGCAGAGGGCGCGGCGCAGGCGGCGGAGGTCGGC-GAGGTGCGGCGGAGGGGATGGTGACGGTGGGGAGATGGACGGAGGAGGGGACCC : 534
 CGCAGGGGGA ACCAGCAAGCAGAGGGCGCGGCGCAGGCGGCGGAGGTCGGC GAGGTGCGGCGGAGGGGATGGTGACGGTGGGGAGATGGACGGAGGAGGGGACCC

 540 * 560 * 580 * 600 * 620 * 640
Hox1_1 : CAAGAAGCGGCGGCTCACCGACGAGCAGGCCGAGATTCTGGAGCTGAGCTTCCGGGAGGACCGCAAGCTGGAGACAGCCCGCAAGGTGTATCTGGCCGCCGAGCTCG : 641
Hox1_30 : CAAGAAGCGGCGGCTCACCGACGAGCAGGCCGAGATTCTGGAGCTGAGCTTCCGGGAGGACCGCAAGCTGGAGACAGCCCGCAAGGTGTATCTGGCCGCCGAGCTCG : 641
Hox1_38 : CAAGAAGCGGCGGCTCACCGACGAGCAGGCCGAGATTCTGGAGCTGAGCTTCCGGGAGGACCGCAAGCTGGAGACAGCCCGCAAGGTGTATCTGGCCGCCGAGCTCG : 642
Hox1_86 : CAAGAAGCGGCGGCTCACCGACGAGCAGGCCGAGATTCTGGAGCTGAGCTTCCGGGAGGACCGCAAGCTGGAGACAGCCCGCAAGGTGTATCTGGCCGCCGAGCTCG : 641
Hox1_95 : CAAGAAGCGGCGGCTCACCGACGAGCAGGCCGAGATTCTGGAGCTGAGCTTGCGGGAGGACCGCAAGCTGGAGACAGCCCGCAAGGTGTATCTGGCCGCCGAGCTCG : 641
Hox1_96 : CAAGAAGCGGCGGCTCACCGACGAGCAGGCCGAGATTCTGGAGCTGAGCTTCCGGGAGGACCGCAAGCTGGAGACAGCCCGCAAGGTGTATCTGGCCGCCGAGCTCG : 641
Hox1_101 : CAAGAAGCGGCGGCTCACCGACGAGCAGGCCGAGATTCTGGAGCTGAGCTTCCGGGAGGACCGCAAGCTGGAGACAGCCCGCAAGGTGTATCTGGCCGCCGAGCTCG : 641
 CAAGAAGCGGCGGCTCACCGACGAGCAGGCCGAGATTCTGGAGCTGAGCTTcCGGGAGGACCGCAAGCTGGAGACAGCCCGCAAGGTGTATCTGGCCGCCGAGCTCG

 * 660 * 680 * 700 * 720 * 740
Hox1_1 : GGCTGGACCCCAAGCAGGTCGCCGTGTGGTTCCAGAACCGCCGCGCGCGCCACAAGAACAAGACGCTCGAGGAGGAGTTCGCGAGGCTCAAGCACGCCCACGACGCC : 748
Hox1_30 : GGCTGGACCCCAAGCAGGTCGCCGTGTGGTTCCAGAACCGCCGCGCGCGCCACAAGAACAAGACGCTCGAGGAGGAGTTCGCGAGGCTCAAGCACGCCCACGACGCC : 748
Hox1_38 : GGCTGGACCCCAAGCAGGTCGCCGTGTGGTTCCAGAACCGCCGCGCGCGCCACAAGAACAAGACGCTCGAGGAGGAGTTCGCGAGGCTCAAGCACGCCCACGACGCC : 749
Hox1_86 : GGCTGGACCCCAAGCAGGTCGCCGTGTGGTTCCAGAACCGCCGCGCGCGCCACAAGAACAAGACGCTCGAGGAGGAGTTCGCGAGGCTCAAGCACGCCCACGACGCC : 748
Hox1_95 : GGCTGGACCCCAAGCAGGTCGCCGTGTGGTTCCAGAACCGCCGCGCGCGCCACAAGAACAAGACGCTCGAGGAGGAGTTCGCGAGGCTCAAGCACGCCCACGACGCC : 748
Hox1_96 : GGCTGGACCCCAAGCAGGTCGCCGTGTGGTTCCAGAACCGCCGCGCGCGCCACAAGAACAAGACGCTCGAGGAGGAGTTCGCGAGGCTCAAGCACGCCCACGACGCC : 748
Hox1_101 : GGCTGGACCCCAAGCAGGTCGCCGTGTGGTTCCAGAACCGCCGCGCGCGCCACAAGAACAAGACGCTCGAGGAGGAGTTCGCGAGGCTCAAGCACGCCCACGACGCC : 748
 GGCTGGACCCCAAGCAGGTCGCCGTGTGGTTCCAGAACCGCCGCGCGCGCCACAAGAACAAGACGCTCGAGGAGGAGTTCGCGAGGCTCAAGCACGCCCACGACGCC

 * 760 * 780 * 800 * 820 * 840 *
Hox1_1 : GCCATCCTCCACAAATGCCACCTCGAGAACGAGGTATGCTTGCTCGCATACACTCACACTGGCTTACATATGGCGCTGCACATCTGCAGTTCCTCTCCGTTCTTGAA : 855
Hox1_30 : GCCATCCTCCACAAATGCCACCTCGAGAACGAGGTATGCTTGCTCGCATACACTCACACTGGCTTACATATGGCGCTGCACATCTGCAGTTCCTCTCCGTTCTTGAA : 855
Hox1_38 : GCCATCCTCCACAAATGCCACCTCGAGAACGAGGTATGCTTGCTCGCATACATTCACACTGGCTTACATATGGCGCTGCACATCTGCAGTTCCTCTCCGTTCTTGAA : 856
Hox1_86 : GCCATCCTCCACAAATGCCACCTCGAGAACGAGGTATGCTTGCTCGCATACACTCACACTGGCTTACATATGGCGCTGCACATCTGCAGTTCCTCTCCGTTCTTGAA : 855
Hox1_95 : GCCATCCTCCACAAATGCCACCTCGAGAACGAGGTATGCTTGCTCGCATACACTCACACTGGCTTACATATGGCGCTGCACATCTGCAGTTCCTCTCCGTTCTTGAA : 855
Hox1_96 : GCCATCCTCCACAAATGCCACCTCGAGAACGAGGTATGCTTGCTCGCATACACTCACACTGGCTTACATATGGCGCTGCACATCTGCAGTTCCTCTCCGTTCTTGAA : 855
Hox1_101 : GCCATCCTCCACAAATGCCACCTCGAGAACGAGGTATGCTTGCTCGCATACATTCACACTGGCTTACATATGGCGCTGCACATCTGCAGTTCCTCTCCGTTCTTGAA : 855
 GCCATCCTCCACAAATGCCACCTCGAGAACGAGGTATGCTTGCTCGCATACA TCACACTGGCTTACATATGGCGCTGCACATCTGCAGTTCCTCTCCGTTCTTGAA

 860 * 880 * 900 * 920 * 940 * 960
Hox1_1 : CATGCTTACTGACAAACATATGGCCAGCTGCTGAGGCTGAAGGAGAGACTGGGAGCGACTGA-CAGGAGGTGCGGCGCCTCAGGTCGGCAGCTGGGAGCCACGGGGC : 961
Hox1_30 : CATGCTTACTGACAAACATATGGCCAGCTGCTGAGGCTGAAGGAGAGACTGGGAGCGACTGAGCAGGAGGTGCGGCGCCTCAGGTCGGCAGCTGGGAGCCACGGGGC : 962
Hox1_38 : CATGCTTACTGACAAACATATGGCCAGCTGCTGAGGCTGAAGGAGAGACTGGGAGCGACTGAGCAGGAGGTGCGGCGCCTCAGGTCGGCAGCTGGGAGCCACGGGGC : 963
Hox1_86 : CATGCTTACTGACAAACATATGGCCAGCTGCTGAGGCTGAAGGAGAGACTGGGAGCGACTGAGCAGGAGGTGCGGCGCCTCAGGTCGGCAGCTGGGAGCCACGGGGC : 962
Hox1_95 : CATGCTTACTGACAAACATATGGCCAGCTGCTGAGGCTGAAGGAGAGACTGGGAGCGACTGAGCAGGAGGTGCGGCGCCTCAGGTCGGCAGCTGGGAGCCACGGGGC : 962
Hox1_96 : CATGCTTACTGACAAACATATGGCCAGCTGCTGAGGCTGAAGGAGAGACTGGGAGCGACTGA-CAGGAGGTGCGGCGCCTCAGGTCGGCAGCTGGGAGCCACGGGGC : 961
Hox1_101 : CATGCTTACTGACAAACATATGGCCAGCTGCTGAGGCTGAAGGAGAGACTGGGAGCGACTGAGCAGGAGGTGCGGCGCCTCAGGTCGGCAGCTGGGAGCCACGGGGC : 962
 CATGCTTACTGACAAACATATGGCCAGCTGCTGAGGCTGAAGGAGAGACTGGGAGCGACTGA CAGGAGGTGCGGCGCCTCAGGTCGGCAGCTGGGAGCCACGGGGC

 * 980 * 1000 * 1020 * 1040 * 1060 *
Hox1_1 : ATCTGTGGATGGCGGACACGCCGCTGGCGCCGTTGGCGTGTGCGGCGGGAGCCCGAGCTCGTCCTTCTCGACGGGAACCTGCCAGCAGCAGCCGGGTTTCAGCGGGG : 1068
Hox1_30 : ATCTGTGGATGGCGGACACGCCGCTGGCGCCGTTGGCGTGTGCGGCGGGAGCCCGAGCTCGTCCTTCTCGACGGGAACCTGCCAGCAGCAGCCGGGTTTCAGCGGGG : 1069
Hox1_38 : ATCTGTGGATGGCGGACACGCCGCTGGCGCCGTTGGCGTGTGCGGCGGGAGCCCGAGCTCGTCCTTCTCGACGGGAACCTGCCAGCAGCAGCCGGGTTTCAGCGGGG : 1070
Hox1_86 : ATCTGTGGATGGCGGACACGCCGCTGGCGCCGTTGGCGTGTGCGGCGGGAGCCCGAGCTCGTCCTTCTCGACGGGAACCTGCCAGCAGCAGCCGGGTTTCAGCGGGG : 1069
Hox1_95 : ATCTGTGGATGGCGGACACGCCGCTGGCGCCGTTGGCGTGTGCGGCGGGAGCCCGAGCTCGTCCTTCTCGACGGGAACCTGCCAGCAGCAGCCGGGTTTCAGCGGGG : 1069
Hox1_96 : ATCTGTGGATGGCGGACACGCCGCTGGCGCCGTTGGCGTGTGCGGCGGGAGCCCGAGCTCGTCCTTCTCGACGGGAACCTGCCAGCAGCAGCCGGGTTTCAGCGGGG : 1068
Hox1_101 : ATCTGTGGATGGCGGACACGCCGCTGGCGCCGTTGGCGTGTGCGGCGGGGGCCCGAGCTCGTCCTTCTCGACGGGAACCTGCCAGCAGCAGCCGGGTTTCAGCGGGG : 1069
 ATCTGTGGATGGCGGACACGCCGCTGGCGCCGTTGGCGTGTGCGGCGGGaGCCCGAGCTCGTCCTTCTCGACGGGAACCTGCCAGCAGCAGCCGGGTTTCAGCGGGG

 1080 * 1100 * 1120 * 1140 * 1160 * 1
Hox1_1 : CAGACGTGCTGGGGCGGGACGATGACCTGATGATGTGCGTCCCCGAGTGGTTTTTAGCATGAATTAGAGTTTATGCTGGCTAAGCTGATAGCAGCGTGGTCGAGTGT : 1175
Hox1_30 : CAGACGTGCTGGGGCGGGACGATGACCTGATGATGTGCGTCCCCGAGTGGTTTTTAGCATGAATTAGAGTTTATGCTGGCTAAGCCGATAGCAGCGTGGTCGAGTGT : 1176
Hox1_38 : CAGACGTGCTGGGGCGGGACGATGACCTGATGATGTGCGTCCCCGAGTGGTTTTTAGCATGAATTAGAGTTTATGCTGGCTAAGCCGATAGCAGCGTGGTCgaGTGT : 1177
Hox1_86 : CAGACGTGCTGGGGCGGGACGATGACCTGATGATGTGCGTCCCCGAGTGGTTTTTAGCATGAATTAGAGTTTATGCTGGCTAAGCCGATAGCAGCGTGGTCGAGTGT : 1176
Hox1_95 : CAGACGTGCTGGGGCGGGACGATGACCTGATGATGTGCGTCCCCGAGTGGTTTTTAGCATGAATTAGAGTTTATGCTGGCTAAGCCGATAGCAGCGTGGTCGAGTGT : 1176
Hox1_96 : CAGACGTGCTGGGGCGGGACGATGACCTGATGATGTGCGTCCCCGAGTGGTTTTTAGCATGAATTAGAGTTTATGCTGGCTAAGCTGATAGCAGCGTGGTCGAGTGT : 1175
Hox1_101 : CAGACGTGCTGGGGCGGGACGATGACCTGATGATGTGCGTCCCCGAGTGGTTTTTAGCATGAATTAGAGTTTATGCTGGCTAAGCCGATAGCAGCGTGGTCGAGTGT : 1176
 CAGACGTGCTGGGGCGGGACGATGACCTGATGATGTGCGTCCCCGAGTGGTTTTTAGCATGAATTAGAGTTTATGCTGGCTAAGC GATAGCAGCGTGGTCGAGTGT

 180 *
Hox1_1 : TTTTTAGCATGAAAT : 1190
Hox1_30 : TTTTTAGCATGAAAT : 1191
Hox1_38 : TTTTTAGCATGAAAT : 1192
Hox1_86 : TTTTTAGCATGAAAT : 1191
Hox1_95 : TTTTTAGCATGAAAT : 1191
Hox1_96 : TTTTTAGCATGAAAT : 1190
Hox1_101 : TTTTTAGCATGAAAT : 1191
 TTTTTAGCATGAAAT
